# Supplementary material for: Phase Diagrams of Polymerization-Induced Self-Assembly Are Largely Determined by Polymer Recombination
Source: Polymers (Basel). 2022 Dec 6;14(23):5331. doi: 10.3390/polym14235331 (PMC9736918; doi:10.3390/polym14235331)
Supplement: Supplementary file 1 [file polymers-14-05331-s001.zip › polymers-2005729-supplementary.pdf]

Supplementary Materials

Phase Diagrams of Polymerization-Induced Self-Assembly Are Largely Determined by Polymer Recombination

S1. Molecular Weight Distributions of ATRP PISA

In this section, we analyze the molecular weight distributions (MWDs) of the studied systems. Black lines in Figure S1 show the MWDs for the systems simulated according to the ATRP algorithm as described in the main text (data for $p_t = 0.05$ are shown, 95% of all chains terminated). We see that all distributions have only one peak, i.e., they are unimodal.

If we look at the reaction probabilities used to simulate ATRP (see the main text), we will see that the ratios of reaction rate constants were captured unrealistically. For example, a typical RDRP process realized in experiments exhibits the following ratios between termination rate constant (k_t), initiation rate constant (k_i), propagation rate constant (k_p), and the rate constants of reactions of activation/deactivation of the active growing chain end (k_{act}, k_{deact}): $k_t/k_i \approx 10^2$, $k_i/k_p \approx 10^{2\pm1}$, $k_{act}/k_{deact} \approx 10^{-7}$.[1] On the other hand, our fast ATRP simulation algorithm has the following ratios of reaction probabilities: $p_t/p_i \approx 10^{-2}$, $p_i/p_p \approx 10^1$, $p_{B,dorm} = 0.9$, $p_{A,dorm} = 0.99$. Here $p_{B,dorm}$ and $p_{A,dorm}$ are equivalent to the $1 - k_{act}/k_{deact}$. As we see, the p_t/p_i ratio is captured incorrectly in our model: the reaction of termination is too slow. Moreover, the fraction of dormant chains is too low compared to the experimental systems. These two factors allow faster simulation of ATRP.

Therefore, we need to check how the aforementioned discrepancies between our model and the experimentally realized ATRP affect ATRP PISA phase diagrams. The "adjustments" of the reaction rate constants used in our simulations may have affected ATRP PISA phase diagrams only by having influence on MWDs. In order to check whether or not this effect was present, we carried out simulations of ATRP PISA with more realistic "slowed down" reactions. We increased the probability of termination by 4.5 times ($p_t = 0.225$) and decreased the propagation probability by 4.5 times ($p_p = 0.0111$). In order to preserve the ratio between the rates of propagation and termination, we needed to increase the fraction of dormant chains in the system, so we set $p_{B,dorm} = 0.995$, $p_{A,dorm} = 0.9995$. Initiation probability p_i was set to $p_i = 0.04$. Therefore, the fractions of dormant chains and the ratios between the reaction probabilities were captured more realistically: $p_t/p_i \approx 10^0$, $p_i/p_p \approx 10^0$

($p_t > p_i > p_p$, as is in the experiments). We carried out simulations with $N_{stp} = 20$ in a simulation box with a size of 60^3 (the box size was decreased to speed up the calculations, as the reaction proceeded very slowly). All other simulation parameters were chosen as described in the main text. MWDs after reaching 100% conversion and 94% fraction of terminated chains are shown in Figure S1 (red lines).

Figure S1 clearly demonstrates that the "fast" ATRP simulation algorithm yields MWDs very similar to the more realistic "slow" method. As a result, we believe that the obtained phase diagrams of ATRP PISA reproduce trends anticipated in real experiments on ATRP PISA.

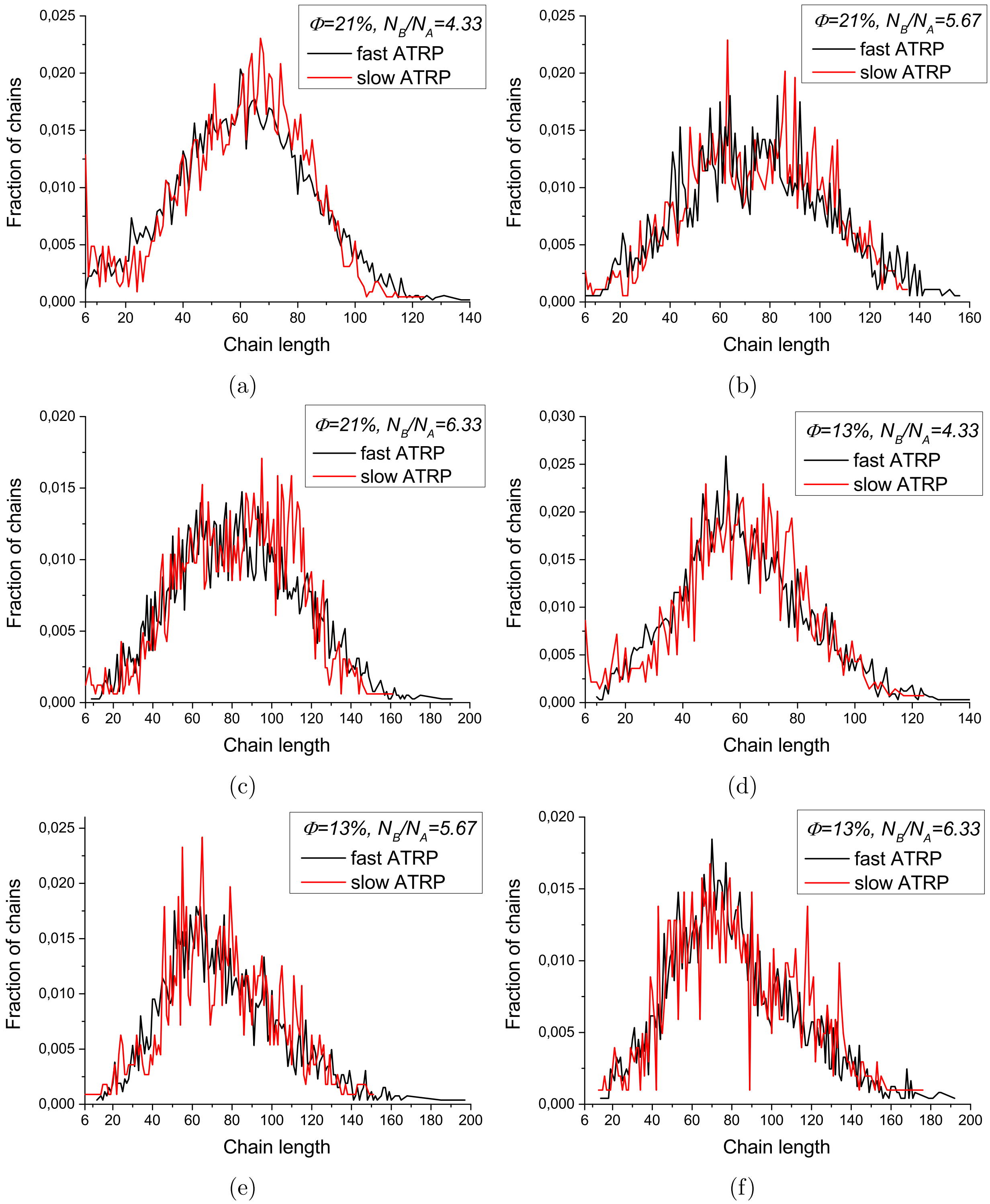

Figure S1: Molecular weight distributions (MWDs) in various studied systems at different polymer volume fractions Φ and different values of N_B/N_A. Black lines represent MWDs in the systems simulated according to the procedure described in the main text ($p_t = 0.05$, 95% of all chains terminated). Red lines represent MWDs in the systems with "slow" chemical reactions (94% of all chains underwent recombination, see Section S1 in Supplementary Materials).

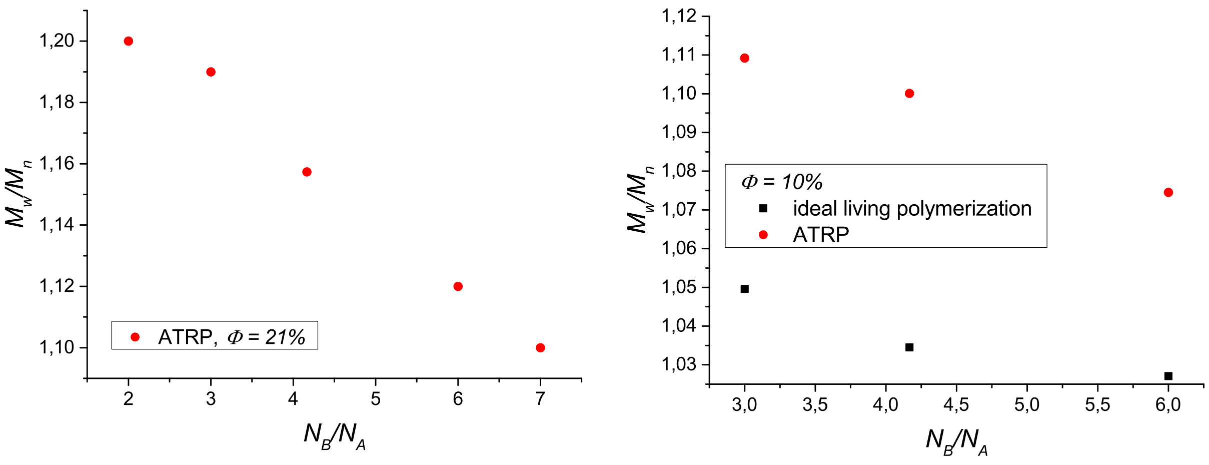

(a) (b)

Figure S2: Dispersity of chains as a function of N_B/N_A values at 100% conversion ($p_t = 0$). Black dots - dispersities achieved during PISA induced by ideal living polymerization at 100% conversion.[2]

S2. Fast Algorithm of ATRP PISA Phase Diagram Calculation

Running the process of ATRP-induced self-assembly long enough is a natural way to obtain equilibrium block copolymer assemblies, and thus, to calculate phase diagrams. However, if the chain dispersity is large enough or polymers have different architecture (for example, diblock copolymers can be mixed with triblock copolymers due to recombination), it becomes unfeasible to simulate this natural evolution of the systems towards equilibrium due to the very large relaxation times observed for such systems. In this section, we describe a method to calculate equilibrium phase diagrams orders of magnitude faster than via the natural system evolution.

First, ATRP PISA was run as described in Methods until 100% conversion and a desired fraction of terminated chains was achieved. After this, simulations were stopped and a structural file obtained. This file was used as an initial structure for a DPD simulation with all parameters chosen as described in Methods except for conservative interaction parameters

a_{ij}. The coefficients a_{ij} were chosen as follows: $a_{AB} = 25.0$, $a_{SB} = 31.21$, $a_{AS} = 25.0$ (i.e., $\chi_{AB} = \chi_{AS} = 0$, $\chi_{SB} = 1.9$). After setting parameters, we ran the DPD simulation without ATRP reaction. During this simulation, we gradually changed the values of certain a_{ij} coefficients to achieve polymer precipitation. In particular, a_{SB} was being increased by 0.01 every 1000 DPD time steps until this parameter reached the value $a_{SB} = 57.7$. Simultaneously, the coefficient a_{AS} was being increased by ~ 0.0012 every 1000 DPD time steps, so a_{AS} reached the value $a_{AS} = 28.27$ by the time a_{SB} reached the value $a_{SB} = 57.7$. After this increase, all simulation parameters were fixed, and the simulation was continued. Therefore, we performed a slow increase of DPD coefficients to make the Flory-Huggins parameters equal to $\chi_{AB} = 0$, $\chi_{SB} \approx 10$, $\chi_{AS} \approx 1$. Such a procedure caused precipitation of polymers in all studied systems into a single spherical aggregate with little or no solvent inside the aggregate. After precipitation, we carried out simulations for additional 2×10^6 DPD time steps to equilibrate the aggregate.

Second, we carried out a DPD simulation using the precipitated and equilibrated structure as an initial file. All DPD coefficients were set as described in Methods for modelling ATRP PISA. In particular, all Flory-Huggins parameters were set to zero except for χ_{AB} and χ_{SB}, which were equal to $\chi_{AB} = \chi_{SB} = 1.9$. During this simulation, the aforementioned precipitate disaggregated, and chains formed a stable morphology after $\propto 10^7$ DPD time steps.

To verify that the algorithm described above yielded equilibrium block copolymer morphologies, we calculated phase diagrams both by the fast algorithm and by natural evolution of systems for $p_t = 0$ (no recombination, only diblock copolymers are present in the system). The comparison between these two phase diagrams is shown in Figure S3.

We see that the lines of transition between cylindrical micelles and vesicles coincided exactly. The transition between the region of spherical/cylindrical micelles coexistence and the region of existence of pure cylindrical micelles determined by the fast algorithm deviated a little from the natural system evolution. We attribute this slight discrepancy to the

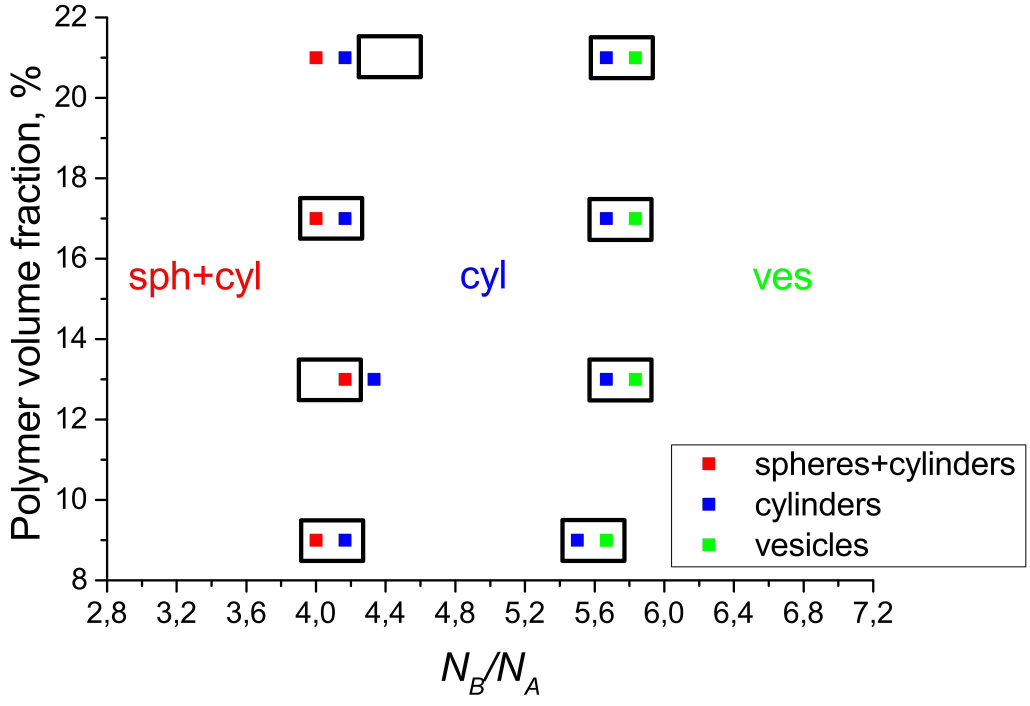

Figure S3: Comparison of the phase diagrams of ATRP PISA without recombination ($p_t = 0$) obtained by the fast algorithm (dots) and by the natural system evolution (black rectangles). Red dots represent the state of coexistence between spherical and cylindrical micelles, blue dots show the state of pure cylindrical micelles, and green dots represent vesicles. Black rectangles show the points of transition determined by long enough simulations of natural system evolution.

statistical deviations and impossibility to determine precisely when the region of coexistence turns into the region of pure cylindrical micelles. Hence, the fast algorithm yielded correct thermodynamically equilibrium phase diagram in the $p_t = 0$ case.

S3. Self-Assembly of Monodisperse ABA-Triblock-Copolymers

In this section, we describe the simulation of self-assembly of monodisperse ABA-triblock-copolymers. We carried out simulations of the transition between cylindrical micelles and vesicles. The method of obtaining equilibrium aggregates was very similar to the ”fast” algorithm employed to calculate other phase diagrams (see Supplementary Materials, Section S2). However, there were some technical differences from that algorithm.

First, a solution of monodisperse ABA-triblock-copolymers was generated artificially, without employing the ATRP simulation. Second, a single aggregate of chains was obtained as follows. Initially, the conservative DPD interaction coefficients a_{ij} were chosen as follows: $a_{AB} = 25.0$, $a_{SB} = 29.0$, $a_{AS} = 29.0$. After this, both a_{AS} and a_{SB} coefficients were being increased simultaneously by 0.03 every 10^4 DPD time steps for 6.4×10^6 DPD time steps. By the end of this simulation stage, all chains formed a single aggregate, and χ values were equal to $\chi_{AB} = 0$, $\chi_{AS} = \chi_{BS} \approx 5.9$. Third, after this aggregate had been obtained, we set the a_{ij} coefficients to $a_{SB} = 57.7$, $a_{AS} = 28.27$, as described in Supplementary Materials, Section S2, and carried out simulations for 2×10^6 DPD time steps. As a result, we obtained a single equilibrated aggregate of ABA-triblock-copolymers, equivalent to the aggregate obtained to calculate the ATRP PISA phase diagrams. Finally, the Flory-Huggins parameters were set to $\chi_{AB} = \chi_{SB} = 1.9$, and simulations proceeded until an equilbrium morphology was obtained. The transition line for ABA-triblock-copolymers almost coincided with the transition line for AB-diblock-copolymers (Figure S4).

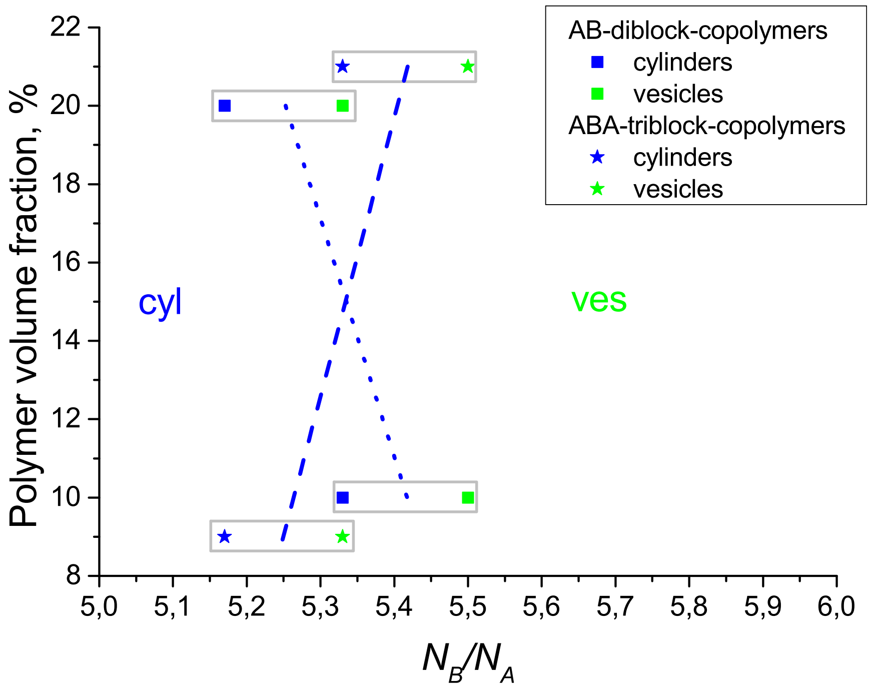

Figure S4: Comparison of the lines of transition between cylindrical micelles and vesicles for monodisperse ABA-triblock-copolymers and AB-diblock-copolymers.

S4. Elucidating the Role of Polymer Concentration in ATRP PISA

In this section, we demonstrate that the polymer concentration by itself has a little influence on the micellar morphology for the systems with $\approx 95\%$ of terminated chains; we believe that the concentration-dependent change of the B-block dispersity, and not the change of concentration itself, led to the difference between cylinder-vesicle transition points at different concentrations in those systems.

To substantiate the aforementioned point, we took the systems at $\Phi = 13\%$, $p_t = 0.05$ (95% of all chains underwent recombination). Three systems were analyzed: (i) $N_B/N_A \approx 6.67$, (ii) $N_B/N_A \approx 6.83$, and (iii) $N_B/N_A \approx 7$. At $\Phi = 13\%$, the system (i) yielded cylinders, while the systems (ii) and (iii) yielded lamellar morphology. For those three systems, we performed polymer precipitation as described in Supplementary Materials, Section S2, and obtained a spherical aggregate. After this, we removed solvent particles from the systems to

make polymer volume fraction equal to $\Phi = 21\%$ and decreased the simulation box size to preserve DPD density of 3 particles per unit volume.

After this, we performed simulations of the disaggregation of this precipitate in all three systems as described in Supplementary Materials, Section S2 (all DPD coefficients were set as for ATRP PISA modeling described in Methods). As a result, we obtained cylindrical micelles for the systems (i) and (ii), and a lamellar morphology for the system (iii) (Figure S5). Therefore, we see that the change of molecular weight distribution is the driving force for the concentration-dependent cylinder-vesicle transition.

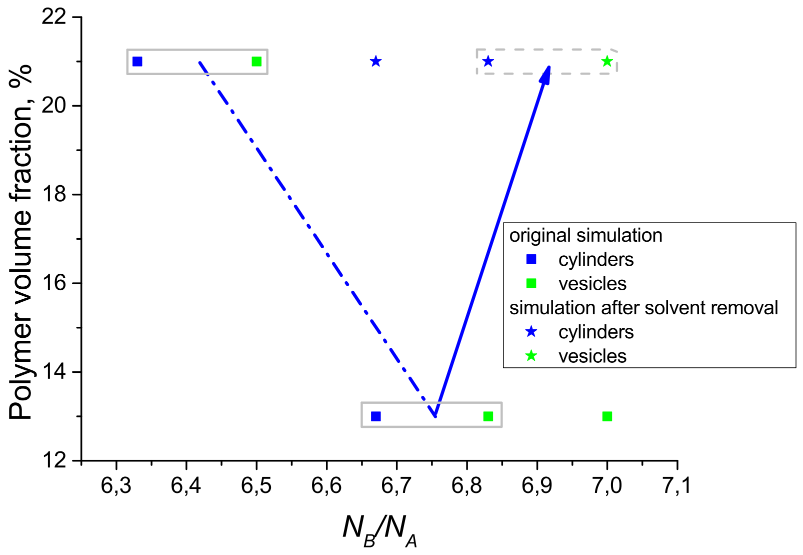

Figure S5: Points of transition between cylindrical micelles and vesicles for the systems with $\approx 95\%$ of terminated chains. Blue dashed-dotted line shows the cylinder-vesicle transition line in the original simulation (Figure 3b in the main text). Blue arrow shows how the transition point shifted after the process of solvent removal described above.

S5. Conformations of the Central B Blocks of ABA Triblocks inside the Micelles

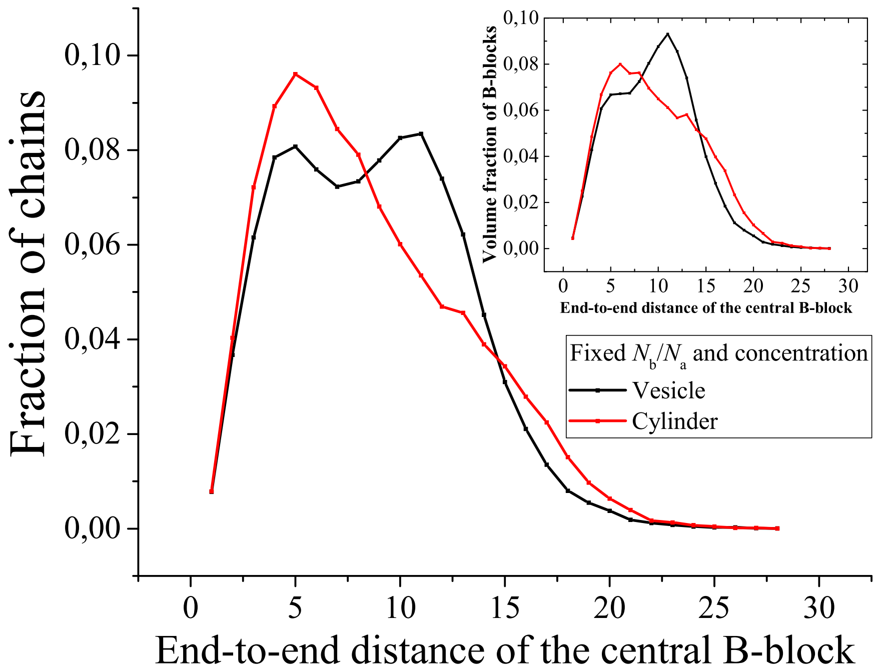

Figure S6: Comparison of the distributions of the end-to-end distances of the central B-blocks of the ABA-triblock-copolymers in the systems at a fixed value of N_B/N_A=6.67 and $\Phi = 21\%$. The vesicle is obtained during standard ATRP PISA routine, while the cylinder was obtained by the solvent removal procedure. Inset shows the same distribution, but for the volume fraction of B blocks instead of the number fraction.

In this section, we compare two systems at a fixed value of N_B/N_A=6.67 and polymer volume fraction of $\Phi = 21\%$. One of those systems was obtained using the standard ATRP PISA routine with 95% of terminated chains (Figure 3 in the main text); the dispersity of the B-block of the ABA copolymers in this system was equal to 1.17. The second system, on the other hand, was obtained by applying the solvent removal process to the system with N_B/N_A=6.67 and $\Phi = 13\%$ as described in Section 4 above; the dispersity of the B-block of the ABA copolymers in this system was equal to 1.23. Different micellar morphologies were observed for these two systems: vesicles for the former (Figure 3b) and cylinders for the latter (Figure S5). We compared the end-to-end distances distribution of the central B-

blocks in these two systems; the results are presented in Figure S6. One can clearly see that the chain ensemble forming the cylinder has a longer tail of chains having larger end-to-end distances. This is especially apparent if we look at the inset which shows that chains with larger end-to-end distances occupy noticeably more space in the micellar cores.

References

(1) Genzer, J. In silico polymerization: Computer simulation of controlled radical polymerization in bulk and on flat surfaces. *Macromolecules* **2006**, *39*, 7157–7169.

(2) Gavrilov, A. A.; Shupanov, R. M.; Chertovich, A. V. Phase Diagram for Ideal Diblock-Copolymer Micelles Compared to Polymerization-Induced Self Assembly. *Polymers* **2020**, *12*, 2599.
